# Lapatinib-Resistant HER2+ Breast Cancer Cells Are Associated with Dysregulation of MAPK and p70S6K/PDCD4 Pathways and Calcium Management, Influence of Cryptotanshinone

**DOI:** 10.3390/ijms26083763

**Published:** 2025-04-16

**Authors:** Jorge Hernández-Valencia, Ruth García-Villarreal, Manuel Rodríguez-Jiménez, Alex Daniel Hernández-Avalos, Ignacio A. Rivero, José Luis Vique-Sánchez, Brenda Chimal-Vega, Angel Pulido-Capiz, Victor García-González

**Affiliations:** 1 Departamento de Bioquímica, Facultad de Medicina Mexicali, Universidad Autónoma de Baja California, Mexicali 21100, Baja California, Mexico; jhernandezv@cinvestav.mx (J.H.-V.); ruth.garcia@uabc.edu.mx (R.G.-V.); srodriguez5@uabc.edu.mx (M.R.-J.); alex.hernandez11@uabc.edu.mx (A.D.H.-A.); brenda.chimal@uabc.edu.mx (B.C.-V.); pulido.angel@uabc.edu.mx (A.P.-C.); 2 Laboratorio Multidisciplinario de Estudios Metabólicos y Cáncer, Facultad de Medicina Mexicali, Universidad Autónoma de Baja California, Mexicali 21100, Baja California, Mexico; 3 Centro de Graduados e Investigación en Química, Tecnológico Nacional de México, Instituto Tecnológico de Tijuana, Tijuana 22510, Baja California, Mexico; irivero@tectijuana.mx; 4 Centro de Ciencias de la Salud Mexicali, Universidad Autónoma de Baja California, Mexicali 21000, Baja California, Mexico; jvique@uabc.edu.mx; 5 Centro de Innovación e Investigación en Salud (CIIS), Universidad Autónoma de Baja California, Mexicali 21000, Baja California, Mexico

**Keywords:** breast cancer HER2+, chemoresistance, lapatinib, calcium homeostasis, cryptotanshinone

## Abstract

Resistance to HER2 tyrosine-kinase inhibitor Lapatinib (Lap) is one of the leading causes of cancer treatment failure in HER2+ breast cancer (BC), associated with an aggressive tumor phenotype. Cryptotanshinone (Cry) is a natural terpene molecule that could function as a chemosensitizer by disturbing estrogen receptor (ERα) signaling and inhibiting the protein translation factor-4A, eIF4A. Therefore, we evaluated Cry dual regulation on eIF4A and ERα. This study aimed to elucidate the underlying mechanisms of Lap chemoresistance and the impact of Cry on them. We generated two Lap-resistant BT474 cell HER2+ variants named BT474^LapRV1^ and BT474^LapRV2^ with high chemoresistance levels, with 7- and 11-fold increases in EC_50_, respectively, compared to BT474 parental cells. We found a PDCD4-p70S6Kβ axis association with Lap chemoresistance. However, a concomitant down-regulation of the RAF-MEK-ERK cell survival pathway and NF-κB was found in the chemoresistant cell variants; this phenomenon was exacerbated by joint treatment of Cry and Lap under a Lap plasmatic reported concentration. Optimized calcium management was identified as a compensatory mechanism contributing to chemoresistance, as determined by the higher expression of calcium pumps PMCA1/4 and SERCA2. Contrary to expectations, a combination of Lap and Cry did not affect the chemoresistance despite the ERα down-regulation; Cry-eIF4A binding possibly dampens this condition. Results indicated the pro-survival eIF4A/STAT/Bcl-xl pathway and that the down-regulation of the MAPK-NF-κB might function as an adaptive mechanism; this response may be compensated by calcium homeostasis in chemoresistance, highlighting new adaptations in HER2+ cells that lead to chemoresistance.

## 1. Introduction

Breast cancer (BC) is the most widespread cancer among women worldwide. Approximately 20% of BC shows overexpression of the oncogenic epidermal growth factor receptor 2 (HER2), which is related to high aggressiveness, poor prognosis, and short survival [1]. Lapatinib (Lap) is an inhibitor that can target the tyrosine kinase domains of HER2 and suppress the phosphorylation and signaling of MAPK and Akt/mTOR oncogenic pathways, inhibiting cancer proliferation and leading to apoptosis. However, most patients develop Lap drug resistance [2].

The Akt/mTOR signaling pathway avoids HER2 blockade and its downstream targets p70S6Kα/β could be involved in Lap resistance [3]. Despite their high degree of structural homology, in BC, p70S6Kα is predominantly involved in regulating cell proliferation, invasion, and metastasis, while p70S6Kβ appears to have a more significant impact on cell death regulation [4]. In this regard, the eukaryotic initiation factor 4F complex (eIF4F) composed of eIF4E-eIF4G-eIF4A is located at the convergence of several pathways involved in cancer, notably the PI3K/Akt/mTOR and potentially the oncogenic STAT1 that promotes the expression of B-cell lymphoma xl (*Bcl-xL*) gene [5,6]. When phosphorylated by mTORC1, the 4EBP proteins cannot bind eIF4E, forming an effective eIF4F complex. mTORC1 is also responsible for the phosphorylation of the p70S6Kα, which phosphorylates the programmed cell death 4 (PDCD4) protein and could release its target eIF4A.

Likewise, the activity of p70S6Kβ could be involved in regulating eIF4A [6]. In this regard, eIF4A’s role is critical, using ATP hydrolysis to unwind RNA secondary structures in the untranslated region (UTR) 5′mRNA [6]. For instance, molecules such as zotatifin increase the affinity between eIF4A and specific polypurine sequence motifs in their 5′UTR mRNA, inhibiting the translation of oncogenic drivers HER2 and FGFR1/2 receptor tyrosine kinases [7].

Although HER2+ tumors are initially treated with trastuzumab combination therapy with taxane and anthracycline [8,9], some patients, especially those in metastatic conditions, may become refractory to treatment. Lap, in combination with capecitabine, represents an alternative for patients with trastuzumab resistance [10]. Moreover, CAR-T cell therapy represents another option [11]. However, the acquisition of chemoresistance to Lap may be a reality; thus, it is necessary to expand the universe of available molecules to be used in combination treatments for chemoresistant cancers. Moreover, considering that the potential chemoresistance development in classical druggable pathways, such as KRASG12C mutations (using *Sotorasib* and *Adagrasib*) [12] or CART-therapy [13], reduces the therapeutic options, then the need arises to look for combined therapies.

For instance, tyrosine kinase inhibitors (lapatinib, neratinib, and tucatinib) and antibody-drug conjugate (trastuzumab deruxtecan: T-DXd) showed good antitumor effects against trastuzumab-resistant cells [14], and the use of PI3K/AKT/mTOR inhibitors such as Copanlisib have been shown to restore lapatinib sensitivity in breast cancer [15]. However, expanding the therapeutic arsenal with new molecules at an affordable cost is necessary. One alternative is using molecules isolated from natural sources and dietary phytochemicals. Cryptotanshinone (Cry), a quinoid diterpene extracted from the root of the medicinal plant *Salvia brandegeei*, has been used to treat several diseases [16].

Moreover, the characterization of Cry is based on the structural features of potential targets; eIF4A and ER are strategically involved in essential cell functions; eIF4A is for selective protein translation, and ER is implicated in cellular proliferation. Both signals are involved in the development of chemoresistance [17,18].

Likewise, Cry shows anticancer and chemosensitizing effects in resistant cells. Cry overcomes cisplatin resistance in lung carcinoma cells (A549 cells) [19], and Cry could induce autophagy in a multidrug-resistant human colon cancer cell line through activation of ROS-p38 MAPK-NF-κB signaling [20]. Likewise, Cry could cause the inhibition of estrogen receptor (ER) in BC cells (ERα) [21], with shared mechanisms that could occur on the chemoresistance in the HER2+ subtype. Additionally, the results of our group suggest Cry binding capability on the eIF4A factor [22,23].

Cells dispose of specialized proteins to maintain cytoplasmic calcium concentration at homeostatic conditions, with the plasma membrane Ca^2+^ ATPases (PMCAs) and the sarcoplasmic/endoplasmic reticulum Ca^2+^-ATPase-2 (SERCA2) pump [24] playing crucial roles in calcium extrusion and fine-tuning [25]. Moreover, a relationship has been proposed for the PMCA2 and HER2 levels in breast cancers; PMCA2 expression is activated in breast cancers, wherein PMCA high tumor levels predict increased mortality [26]. This condition could extend to other PMCA variants, such as PMCA1/4, with implications for targets like SERCA2. Therefore, these mechanisms could be overactivated in HER2 breast cancer and favor the development of chemoresistance.

In the present study, we generated Lap highly resistant HER2+ breast cancer variants, BT474^LapRV1^ and BT474^LapRV2^. This chemoresistance phenomenon was associated with a greater capacity for colony formation under treatment with a Lap plasma concentration equivalent to that reported in patients. Although the Cry treatment inhibited the expression of ERα, cell variants showed a resistant behavior to Cry. This resistance is probably due to a Cry-eIF4A binding mechanism. We identified as potential Lap resistance modulators the crosstalk among the ERα and PDCD4-mTOR-p70S6Kβ axis and the down-regulation of the MAPK/ERK pathway. We suggest that cytoplasmic calcium concentrations in HER2+ cells play an essential role in their chemoresistance phenotype, possibly mediated by the calcium pumps PMCA1/4 and SERCA2.

## 2. Results

### 2.1. Characterization of Lap-Resistant Cell Variants

**Figure 1:**
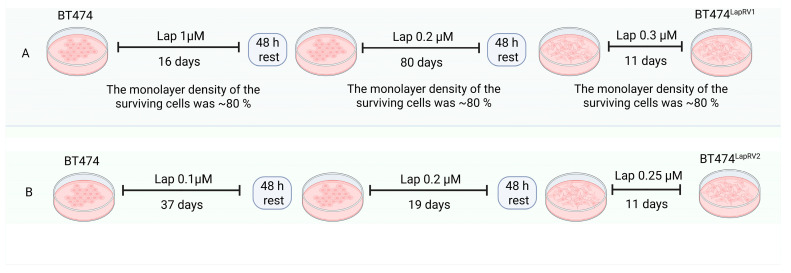
Schematic representation of the generation of Lap-resistant BT474^LapRV1^ and BT474^LapRV2^ cells. (**A**) Process for generation of variant denominated BT474^LapRV1^ by stimulation with high lapatinib dose (1 µM) and later a gradual reduction. (**B**) Variant BT474LapRV2 was generated with an increased range stimulation of lapatinib (0.1–0.25 µM).

We established a Lapatinib-resistant model of HER2+ BC in vitro by treating BT474 cells with a Lap high concentration of 1 µM for 16 days, followed by a gradual increase for 91 days from 0.2 µM Lap to 0.3 µM Lap; this generated a variant referred to as BT474^LapRV1^. In the second scheme, cell cultures were treated under an increasing Lap dose (0.1–0.25 µM) for 67 days, obtaining BT474^LapRV2^ cell variant (Figure 1) (Materials and Methods, Section 4.3). Both variants were maintained for several weeks under a 0.3 µM Lap maintenance dose, which is close to the reported plasma concentrations of Lapatinib-treated patients [27], to emulate the physiological conditions of breast cancer patients.

**Figure 2:**
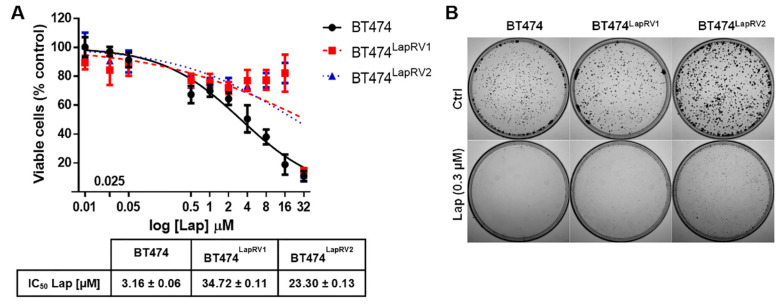
Characterization of Lapatinib-resistant BT474 cell variants. (**A**) Cell viability was evaluated by the MTT assay; BT474 (parental), BT474^LapRV1^, and BT474^LapRV2^ cells were treated with increasing concentrations of Lap for 48 h. Each data point is the mean of three independent experiments ± SD. IC50 values for Lap treatment. (**B**) Colony formation assay of parental and Lap-resistant BT474^LapRV1^ and BT474^LapRV2^ cells seeded in 20 mm plates and treated with Lap (0.3 μM); images correspond to MTT-stained cells.

Based on previous reports, we selected the concentrations in a dose-dependent manner for Lap treatments and the calculation of the IC50 in our parental BT474 cell line. Subsequently, we used the same Lap concentrations in Lap-resistant variants to analyze the dose–response effect and obtain the IC50. On the other hand, because there are no previous reports on the BT474 cell line with Cry treatment, we started with low concentrations (from 1 to 20 µM) in attempting to find antiproliferative effects in a dose-dependent manner. Our resistance-inducing treatments resulted in approximately 7- and 11-fold IC50 increases in BT474LapRV1 (34.72 μM) and BT474LapRV2 (23.30 μM), compared to BT474 parental cells (3.16 µM) (Figure 2A).

As a strategy for characterizing the impact of concentrations reported in the plasma of patients treated with 1200 mg of Lap, with a median peak-plasmatic Lap concentration range between 0.3 and 2.1 µM [27], we used the Lap 0.3 µM as the reference concentration and the maintenance concentration to which cells preserve their resistant phenotype in the long term. Then, a colony formation assay was used to evaluate the stability of Lapatinib resistance and proliferation capability. BT474, BT474^LapRV1^, and BT474^LapRV2^ cells were incubated continually in the presence of Lap (0.3 µM) for 21 days (Figure 2B). The BT474^LapRV1^ and BT474^LapRV2^ cells maintained their resistant phenotype even after extensive Lap treatment but showed a decreased cell proliferation compared with drug-free cells, evidenced by the smaller size of their colonies. We adapted the assay, and the sample processing was performed using MTT staining to evaluate the mitochondrial activity [28]. Results indicated the establishment of Lapatinib-resistant BT474^LapRV1^ and BT474^LapRV2^ cell variants.

### 2.2. Chemoresistance in BT474 Cells Overcomes the Inhibition of HER2 Signaling

**Figure 3:**
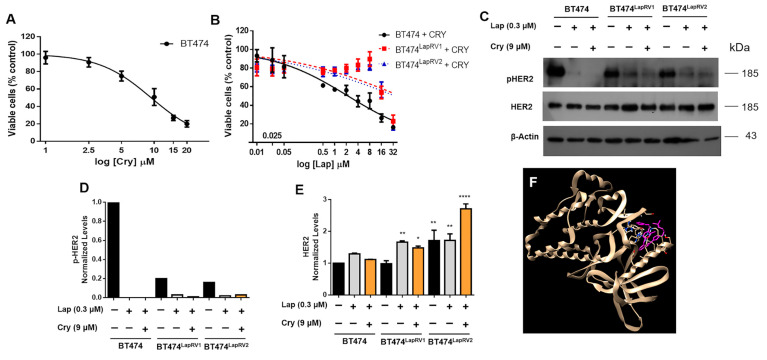
Cryptotanshinone (Cry) maintains chemoresistance in Lapatinib-resistant BT474 cell variants. Cell viability tested by MTT assay; (**A**) Parental cells (BT474) were treated with increasing Cry concentrations (0–20 μM) for 48 h; (**B**) Parental, BT474^LapRV1^, and BT474^LapRV2^ cells were incubated with increasing Lap concentrations and 9 μM Cry for 48 h. Each data point is the mean of three independent experiments ± SD. (**C**) Immunoblot analyses of HER2 and p-HER2 in cells treated with 0.3 μM of Lap and Cry (9 µM) in cell variants. Densitometry analysis of p-HER2 (**D**) and HER2 (**E**) after β-actin normalization; black bar without treatment; grey bar Lap treatment; orange bar Lap plus Cry treatment. Results are presented as the mean of three experiments ± SD. * *p* < 0.05; ** *p* < 0.01; **** *p* < 0.001. (**F**) Docking of lapatinib in the HER2 tyrosine kinase domain (PDB 3PP0). HER2 is shown in beige and lapatinib in magenta.

Given previously observed results of Cry on the multidrug-resistant human colon cancer cell line SW620/Ad300 [20] and its possible effects on eIF4A observed by our group [22], we set out to determine Cry’s anticancer and chemosensitizing effect on BT474 cells and the Lap-resistant variants. Initially, we determined the IC_50_ of Cry in parental cells; results indicate an IC_50_ of 9.34 μM (Figure 3A). Therefore, the potential chemosensitizing effect of Cry was evaluated under a scheme of 9 µM with increasing doses of Lap (0–32 µM) for 48 h to analyze the possible chemosensitizing effect of Cry. As shown in Figure 2B, Cry only sensitized parental cells, reducing the Lap IC_50_ to 2.20 μM (Table 1). However, in Lap-resistant cells BT474^LapRV1^ and BT474^LapRV2^, the Lap IC_50_ increased to 48.28 μM and 36.04 μM, respectively (Figure 3B), compared to 34.7 and 23.3 µM in the absence of Cry treatment (Table 1).

To understand the mechanisms of chemoresistance registered in BT474 variant cells, we determined HER2 expression and phosphorylation levels under a Cry plus Lap combination treatment. First, we demonstrated a reduction in pHER2 in BT474^LapRV1^ and BT474^LapRV2^ regarding parental cells under basal conditions (Figure 3C–E). As a control of the Lapatinib pharmacological effect, we found that HER2 phosphorylation (Tyr^1248^) was suppressed under Lap and Lap plus Cry treatment in parental and resistant cells, indicating a sustained target inhibition (Figure 3C,D). Cry treatment induced a subtle reduction in the expression of HER2 in parental cells and, interestingly, a higher expression in BT474LapRV1 and in BT474LapRV2 cells (Figure 3C–E). Given HER2’s autophosphorylation activity, we explored Lapatinib binding to its kinase domain and found binding in a possible allosteric site, including residues Thr^759^, Ala^763^, and Glu^766^ (Figure 3F). Then, we broadened the analysis to characterize the resistance mechanisms to Lapatinib and identify the molecular determinants associated with the mTOR-p70S6K axis.

### 2.3. The Axis p70S6Kβ-PDCD4 Is Activated in Chemoresistant Cell Variants

**Figure 4:**
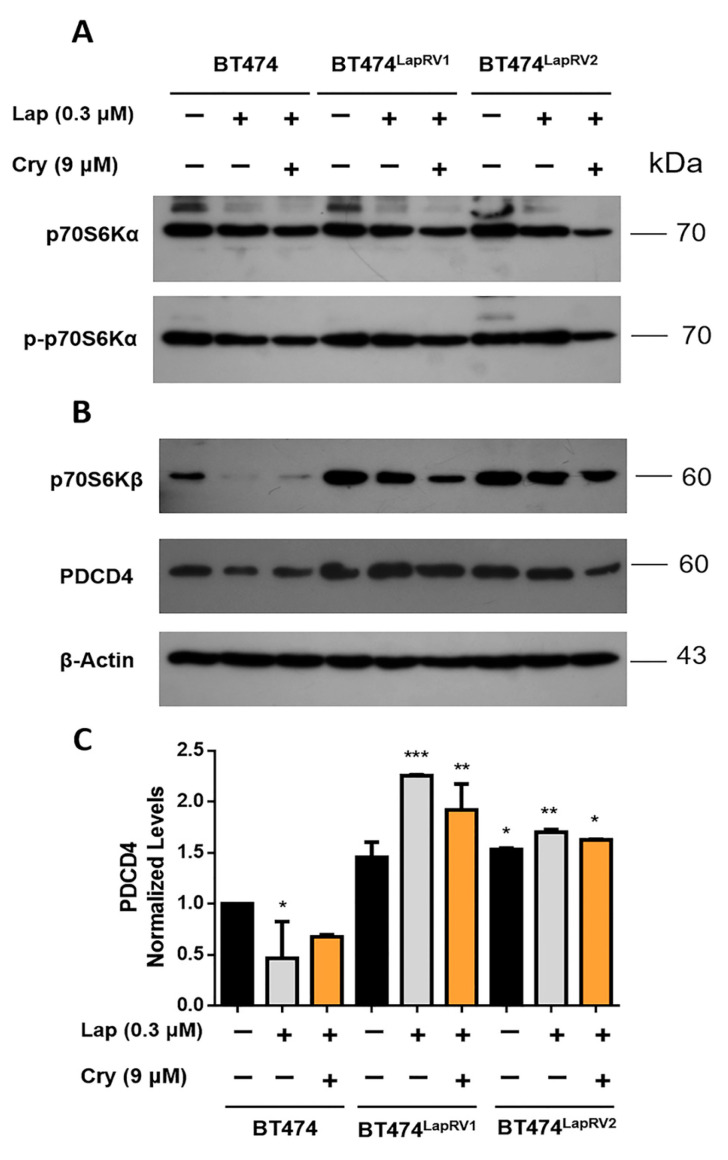
The axis p70S6K-PDCD4 is activated in chemoresistant cells. Chemoresitant cell variants BT474^LapRV1^, BT474^LapRV2^, and BT474 cells were treated under the scheme of Lap (0.3 µM) and Cry (9 µM). Western blot characterization of p70SK6α and p-p70SK6α (**A**), p70SK6β, and PDCD4 (**B**). (**C**) Densitometry analysis of PDCD4; black bar without treatment; grey bar Lap treatment; orange bar Lap plus Cry treatment; results are reported as the mean ± SD (*n* = 3) and expressed as normalized levels against β-actin. * *p* < 0.05; ** *p* < 0.01; *** *p* < 0.001.

Employing the combination treatment with Lap (0.3 µM) and Cry (9 µM), we characterized p70SK6α and its phosphorylated form p-p70SK6α, associated with mTOR activation (Figure 4A). Under the Cry treatment, activation showed a striking reduction in parental, BT474^LapRV1^, and BT474^LapRV2^ cells. This phenomenon corresponded with modifications in HER2 modulation (Figure 4A).

Basal p70S6Kβ expression was higher in BT474^LapRV1^ and BT474^LapRV2^ cells when compared to parental BT474 (Figure 4B). However, Lap and Lap plus Cry treatments induced a higher inhibition of p70S6Kβ expression in BT474 cells, but still a significant expression in both Lap-resistant cell variants was registered (Figure 4B). The tumor suppressor PDCD4 was slightly upregulated in BT474^LapRV1^ and BT474^LapRV2^ cells under Lap and Lap plus Cry treatments, whereas BT474 cells displayed a slight PDCD4 diminution in response to treatments (Figure 4B,C), although PDCD4 levels always remained high in chemoresistant variants. We have described the critical role of PDCD4 in TNBC chemoresistant cells and its potential regulation of eIF4A [22]. The higher expression of PDCD4 and p70S6Kβ could be associated with chemoresistance to Lap, with potential regulation of the eIF4A.

### 2.4. The Role of eIF4A in Chemoresistance and Its Modulation by Crytotanshinone

**Figure 5:**
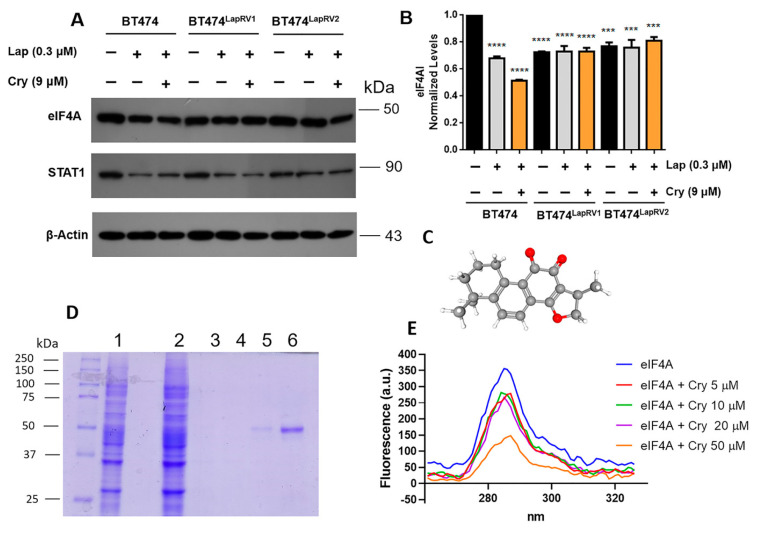
eIF4A Cry treatment. (**A**) Characterization of eIF4A and STAT1 in parental and resistant cells treated with Lap (0.3 μM) and joint Cry (9 µM) for 24 h. (**B**) Densitometric analysis of eIF4A after β-actin normalization. Results are presented as the mean of three independent experiments ± SD. *** *p* < 0.001; **** *p* < 0.0001. (**C**) Structure of terpene molecule Cryptotanshinone. (**D**) eIF4A purification. Lane 1: total lysate; Lane 2: total lysate throughout Ni-NTA column; Lane 3: eluate recovery 1; Lane 4: eluate recovery 2; Lane 5: eluate recovery 3; Lane 6: eluate recovery 4. (**E**) eIF4A Cry binding fluorescence assay.

To explain the compensatory mechanisms that chemoresistant cells have adapted, we characterized the eukaryotic initiation factor eIF4A, associated with the p70S6K-PDCD4 axis. We analyzed parental and resistant cells treated with Lap (0.3 µM) and Cry (9 µM). As shown in Figure 4, Lap and Cry treatment promoted the primary reduction in eIF4A levels in BT474 cells and, furthermore, there is an association between STAT1 levels and the expression of eIF4A (Figure 5A,B). Interestingly, despite the combined Lap and Cry treatment, only a slight reduction in STAT1 levels was observed in BT474^LapRV2^ cells. In this regard, a connection between eIF4A activity assessed by STAT1 expression has been described [29].

Moreover, by focusing on the characterization of eIF4A and the impact on PDCD4, our data suggest that when using an siRNA strategy, eIF4A down-regulation was associated with PDCD4 expression; this association was more evident in the BT474^LapRV2^ variant (Figure S1). This condition strengthens our proposal regarding the role of eIF4A.

Considering the potential STAT1 dependence of eIF4A and the Cry-induced decrease in eIF4A expression, we assayed the interaction of Cry with purified eIF4A (Figure 5C–E). A Cry dose-dependent fluorescence emission decrease in eIF4A associated with the induction of the quenching phenomena was observed (Figure 5D,E), in accordance with our previous report [22]. Moreover, Cry could show a dual role, consistent with eIF4A and the ERα pathway regulation.

### 2.5. Cryptotanshinone Modulates the Estrogen Receptor (ERα)

Preclinical assays have shown an increase in the expression of the ERα and antiapoptotic genes in chemoresistant cells under anti-HER2 therapy [30,31], suggesting a connection between these signaling pathways.

**Figure 6:**
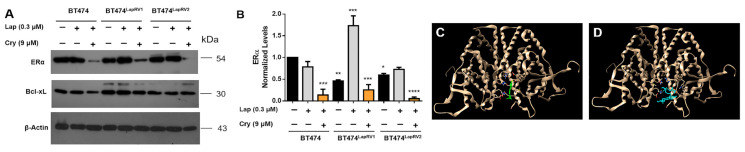
Cry induces estrogen receptor (ERα) down-regulation. (**A**) Expression levels of ERα and Bcl-xL under joint treatment of Lap (0.3 µM) and Cry (9 µM) in BT474, BT474^LapRV1^, and BT474^LapRV2^ cells. (**B**) Densitometry analysis of ERα; black bar without treatment; grey bar Lap treatment; orange bar Lap plus Cry treatment; results are reported as the mean ± SD (*n* = 3); * *p* < 0.05, ** *p* < 0.01, *** *p* < 0.001 and **** *p* < 0.0001 concerning the control. (**C**) Docking experimentation of Cry in the ER structure (PDB ID: 3ERT). ER is shown in beige, and Cry in green. (**D**) Docking of Fulvestrant in the ER structure. ER is shown in beige and fulvestrant in blue.

We characterized ERα expression under Cry and mixed Lap plus Cry treatments. Expression levels in the resistant BT474^LapRV1^ and BT474^LapRV2^ were diminished when compared to BT474 variant cells (Figure S2). In this regard, Lap treatment did not substantially reduce ERα levels (Figure 6A,B) and induced an ERα expression increase in BT474^LapRV1^. In contrast, under the concomitant treatment of Lap (0.3 μM) and Cry (9 μM), we identified a substantial reduction in ERα levels in all three cellular variants (Figure 6A,B), a phenomenon that corresponded with the Cry inhibitory capacity on ERα. Despite modification in HER2 and ERα down-regulation induced by Lap and Cry treatment, the cellular viability was not affected in resistant variants (Figure 3), suggesting high levels of chemoresistance. However, we were unable to detect matrix metalloproteinase-9 (MMP-9) activity in the supernatant media in the cellular variants or an effect due to concomitant treatments (Figure S3). In addition, we identified the increased expression of the anti-apoptotic protein Bcl-xL in BT474^LapRV1^ and BT474^LapRV2^ variants regarding parental cells (Figure 6A). These results suggest that Bcl-xL reinforces the protective responses, as a possibly compensatory anti-apoptotic pathway, in addition to other adaptive mechanisms.

Later, based on molecular docking simulations, we characterized the binding site of Cry on ERα structure; we selected the PDB structure 3ERT for the assays. Our results suggest the Cry interaction is located in the Ligand Binding Domain (LBD) of the ERα, which covers the region of residues 302–595, the same interaction region as estradiol, indicating potential competition for the estrogen receptor’s LBD. In a complementary way, we characterized the molecule fulvestrant as a molecule of reference, a drug used in anti-ERα therapy, promoting ER proteasomal degradation. Potential fulvestrant binding behavior is within the same region as the Cry binding (residues Glu_380_, Glu_423_, Lys_420_, and Met_522_) (Figure 6C,D and Figure S4). The decline of ERα levels under Cry treatment suggests a mechanism similar to that exerted by fulvestrant; although, compensatory mechanisms could be activated.

### 2.6. Dysregulation of Survival Pathways in Chemoresistance Variants

**Figure 7:**
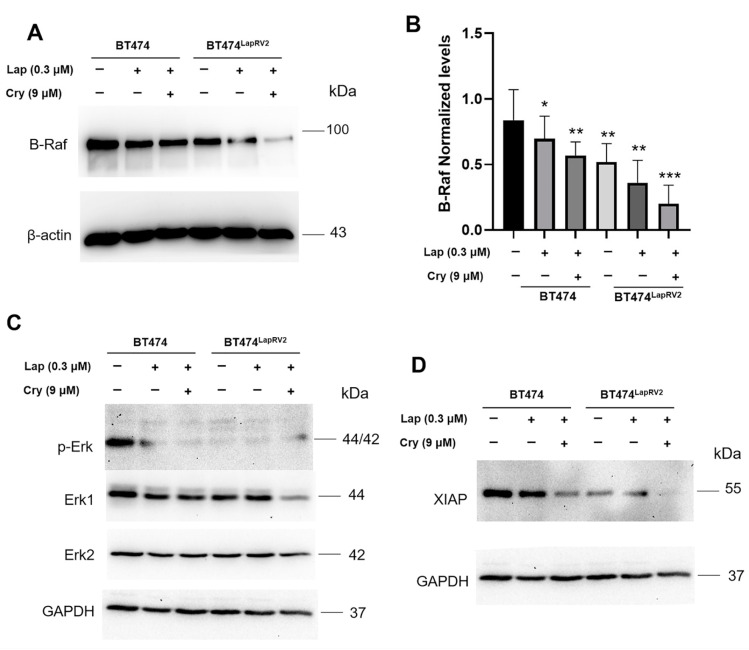
Dysregulation of survival pathways RAF-MEK-ERK in chemoresistance variants. (**A**) Under the scheme of Lap (0.3 µM) and Cry (9 µM) treatment, Western blot of B-Raf in BT474 and BT474^LapRV2^ cells. (**B**) Densitometry analysis of B-Raf; results are reported as the mean ± SD (*n* = 3) and expressed as fold change concerning loading control; * *p* < 0.05, ** *p* < 0.01, and *** *p* < 0.001. Bar colors represent the several treatments. Under the same scheme of Lap (0.3 µM) and Cry (9 µM) treatments, the characterization of pERK1, ERK1, and ERK2 (**C**), as well as XIAP (**D**). β-actin and GAPDH were used as loading controls.

The RAS-regulated RAF-MEK1/2-ERK1/2 signaling pathway is frequently deregulated in human cancers [32]; however, its role in chemoresistance HER2+ breast cancer is not described. We focused on characterizing the B-Raf target (Figure 7A). Nevertheless, for operational reasons, we concentrated on BT474^LapRV2^ cells in this experimental strategy. Contrary to our expectations, under basal conditions, BT474^LapRV2^ variant cells showed lower expression levels than BT474 cells; moreover, treatment of Lap and Lap plus Cry reduced expression levels in both BT474 and BT474^LapRV2^ cells (Figure 7A,B).

In an attempt to characterize the pathway downstream of the canonical MAPkinase pathway, we evaluated the Erk1 and Erk2 expression and the phosphorylation Tyr^204^ (Figure 7C); the results indicated a significant reduction in the Erk1 expression and the down-regulation in phosphorylation in BT474^LapRV2^ concerning BT474 cells under basal conditions (Figure 7C). Moreover, this phenomenon was evident under joint treatment of Lap and Cry (Figure 7C). In the case of Erk2, only under the joint treatment of Lap and Cry did we register a down-regulation of Erk2 in BT474^LapRV2^ cells. Therefore, under basal conditions, this MAPkinase pathway is less active in chemoresistance cells, suggesting the triggering of other compensatory mechanisms.

We focused on the potential adaptive mechanisms; results indicated an unexpected response, a down-regulation in the anti-apoptotic gene XIAP levels under normal conditions without treatment in BT474^LapRV2^ cells regarding parental BT474 cells. This response was exacerbated under Lap and Cry treatment for both cellular variants (Figure 7D). In this regard, PDCD4 could bind to the internal ribosome entry site (IRES) elements of the XIAP mRNA and repress their translation [33], evidence that supports our results connected with PDCD4 expression (Figure 4B).

Based on the data, the RAF-MEK-ERK pathway must be strategically down-regulated; and novel adaptations for escaping cell death could be triggered. In this regard, anti-apoptotic mechanisms and calcium regulation maintain an interdependent regulation [34].

### 2.7. Chemoresistance Is Associated with Optimized Calcium Management

**Figure 8:**
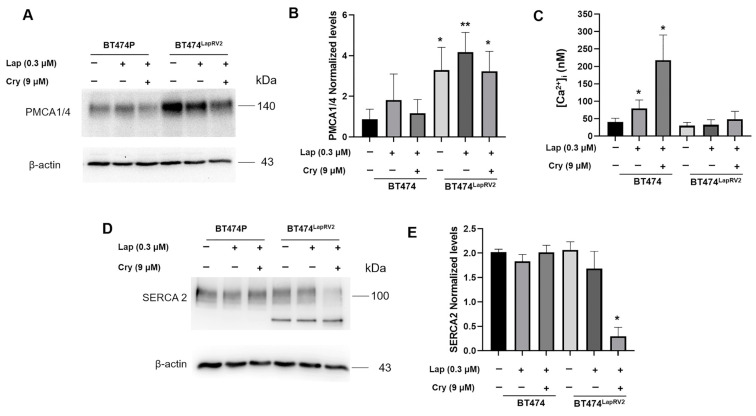
Chemoresistance is associated with optimized calcium management. (**A**) Effect of joint treatment Lap (0.3 µM) and Cry (9 µM) on PMCA1/4 expression in BT474 and BT474^LapRV2^ cells; (**B**) under the same scheme, densitometry analysis of PMCA1/4, results are reported as the mean ± SD (*n* = 3) and expressed as fold change regarding loading control; * *p* < 0.05, ** *p* < 0.01 with regard to control. (**C**) Quantification of intracellular [Ca^2+^] under Lap and Cry treatments in BT474 and BT474^LapRV2^ cells. * *p* < 0.05 with regard to control. Under the same conditions, characterization of SERCA2 by Western blot (**D**), and densitometry analysis of SERCA2 (**E**), results are reported as the mean ± SD (*n* = 3) and expressed as fold change with regard to loading control; * *p* < 0.001 with respect to control. Bar colors represent the different treatments in (**B**,**C**,**E**).

Data from our previous works suggest cells under lipotoxicity and endoplasmic reticulum stress promote adaptive mechanisms in intracellular calcium disposal [35], maintaining cell survival pathways. On this matter, we characterized the effect of concomitant treatment of Lap (0.3 μM) and Cry (9 μM) on the expression of calcium pump PMCA1/4 in BT474 and BT474LapRV2 cells (Figure 8A,B). Results suggested the induction of an adaptive response to calcium disposal, considering the substantial PMCA1/4 increasing expression in chemoresistant BT474^LapRV2^ cells regarding BT474 under basal conditions. Moreover, in BT474^LapRV2^ cells, the levels were only slightly reduced under joint treatment of Lap and Cry (Figure 8A,B).

Therefore, we focused on the impact on intracellular calcium concentrations (Figure 8C), employing the fluorescent probe Fura2-AM; results indicated a substantial increase in calcium levels in BT474 cells under Lap treatment; this phenomenon was exacerbated under the joint treatment of Lap and Cry. Notwithstanding, this response was not found in BT474^LapRV2^ cells (Figure 8C), wherein calcium levels were maintained in a range of 30–50 nM under the treatment of Lap, and only a slight increase in Lap plus Cry treatment was registered (Figure 8C).

To expand the description of cellular responses for the chemoresistant adaptations in calcium management, we focused on the endoplasmic reticulum SERCA2 pump. We characterized the effect of joint treatment of Cry and Lap on SERCA2 expression (Figure 8D,E). The results suggest a differential regulation among PMCA1/4 and SERCA2 expression; for instance, given a lower expression of PMCA1/4 in BT474 cells, the expression levels of SERCA2 remained constant despite the Lap and Lap plus Cry treatments (Figure 8D,E). In the case of BT474^LapRV2^ cells, while treatment with Lap did not affect the expression of this calcium pump, co-treatment with Cry induced a significant reduction in this pump (Figure 8D,E), managing to preserve calcium homeostasis.

### 2.8. NF-κB Pathway and Connection with Chemoresistance

Moreover, an association between calcium levels, PMCA4 regulation, and NF-κB has been proposed in TNBC cells [36]. In addition, Cry could induce autophagy in a multidrug-resistant human colon cancer cell line, by activating ROS-p38 MAPK-NF-κB signaling [20]. Therefore, we continued characterizing the PMCA1/4 implications for NF-κB expression and chemoresistance.

**Figure 9:**
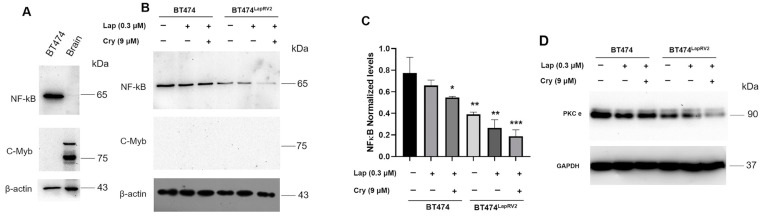
NF-κB down-regulation is associated with chemoresistance. (**A**) Expression of the targets NF-κB and C-Myc in BT474 cells and brain tissue. (**B**) Effect of the Lap (0.3 µM) and Cry (9 µM) joint treatment on the expression of NF-κB and C-Myc in BT474 and BT474^LapRV2^ cells; (**C**) Under the same scheme, densitometry analysis of NF-κB, results are reported as the mean ± SD (*n* = 3) and expressed as fold change regard to loading control; * *p* < 0.05, ** *p* < 0.01, and *** *p* < 0.005 with regard to control. (**D**) Detection of PKCε under Lap and Cry treatments in BT474 and BT474^LapRV2^ cells. β-actin and GAPDH were used as a loading control. Bar colors represent the different treatments.

In the first instance, we characterized the expression of NF-κB in BT474 cells and controls such as mouse brain tissue; we identified the expression of NF-κB only in BT474 cells (Figure 9A). This allowed us to extend the characterization of the influence of the joint treatment of Lap and Cry on both HER2 cellular variants. The results indicated a diminution in NF-κB levels in basal conditions among BT474 and BT474^LapRV2^ cells (Figure 9B,C); moreover, the reduction in NF-κB expression was more pronounced under Lap and Cry treatment, mainly in BT474^LapRV2^ cells (Figure 9B,C). In one report, the inhibition of the NF-κB pathway protects against cell apoptosis and inflammation [37]. Results suggest that NF-κB could be down-regulated as a cellular strategy to maintain cell viability.

The NF-κB signaling activation is mediated by overexpression of transcription factor c-Myb, leading to enhanced proliferation, invasion, and cisplatin resistance in ovarian cancer cells [38]. Under our experimental conditions, we evaluated the potential association with C-Myb; however, the expression levels of this target were not detectable. In this case, we used brain tissue as a control of C-Myb (Figure 9A,B). Moreover, a potential explanation for the regulation of the NF-κB is through isoforms of protein kinase C (PKC); specifically, the knockdown of both PKCε and PKCε has been associated with the decreased NF-κB activity [39]. Based on our results, we identified an association among the levels of the NF-κB with the PKCε expression, in both BT474^LapRV2^ and BT474 cells (Figure 9D).

## 3. Discussion

The values obtained of the Lap IC_50_ of BT474^LapRV1^ and BT474^LapRV2^ (Figure 2A) were comparable with previous reports. For example, Liu et al. reported an IC_50_ = 3.0 µM, and Zhang et al. reported an IC_50_ = 27.02 [40,41]. Based on our MTT assays and the colony formation assay results, resistant cells maintained higher chemoresistance and mitochondrial activity than parental cells and demonstrated a slow proliferation rate under Lap treatment (Figure 1). To extrapolate the effect on parental- and resistant-cell variants within the median peak-plasmatic concentration of Lap (0.3–2.1 µM) reported in patients receiving 1200 mg of Lap [27], we performed the experiments using Lap 0.3 μM, an equivalent concentration. The experiments revealed morphological and functional differences between chemoresistant cell variants and parental cells.

To characterize the mechanism of Lap resistance and the pathways regulated by Cry, we treated parental and resistant cells with Lap 0.3 µM and Cry IC_50_ (9 µM) under acute stimuli for 24 h. Our results showed down-regulation of ERα, which was one of the primary adaptive mechanisms of Lap resistance in BT474^LapRV1^ and BT474^LapRV2^ variants, followed by Bcl-xL adaptation (Figure 3 and Figure 6). Remarkably, Cry treatment inhibited ERα expression; however, this did not reverse the chemoresistance to Lap. (Figure 6A–D). Molecular docking simulations showed that Cry could have a similar mechanism as fulvestrant over the ERα, consistent with the decrease in ER levels. Despite the Lap-induced reduction in cellular viability, our conditions suggest that Cry treatment could induce an adaptive regulation of some elements of the mTOR pathway, promoting an increase in IC50.

Other reports have associated the over-regulation of intracellular kinases such as Akt, mTOR, and p70S6K with the development of chemoresistance [42,43]. p70SK6 comprises p70S6Kα and p70S6Kβ isoforms; p70S6Kα is a kinase that has been suggested to promote cell survival and protein translation [44], while 70S6Kβ appears to have a more significant impact on cell death regulation [4]. In the chemoresistant BT474^LapRV1^ and BT474^LapRV2^, p70S6Kα and p-p70S6Kα showed higher levels than the parental cells (Figure 4); however, joint treatment Lap plus Cry induced their down-regulation. Notwithstanding, data suggest that the increment of p70S6Kβ and PDCD4 are critical targets in chemoresistant cell pathways. Indeed, PDCD4 is highly expressed under basal conditions; furthermore, the treatment of Lap and Lap plus Cry maintained its expression (Figure 4).

Our data indicated a direct link between eIF4A and PDCD4 according to siRNA results. The results suggest the induction of crosstalk of the PDCD4-eIF4A pathway in the resistant cells and modulation by Cry treatment with implications on ERα. In this regard, in aromatase inhibitor-resistant BC cells, HER2 activation could promote the reduction in PDCD4 by activating MAPK, AKT, and miR-21 targets [45]. In contrast, in variants BT474^LapRV1^ and BT474^LapRV2^, under basal conditions without treatment, the HER2 activation decreased and was associated with an increment in PDCD4 expression.

Likewise, eIF4A modifications were registered under Lap and Lap plus Cry treatments. In the parental cells and variants BT474^LapRV1^ and BT474^LapRV2^, the joint treatment of Lap and Cry induced a significant effect on the expression levels of *STAT1*, a gene dependent on the eIF4A (Figure 5A,B). In this regard, our evidence suggested the Cry-binding capability of eIF4A (Figure 5). Indeed, polypurine motifs are enriched in the 5′-UTRs of oncogenic drivers such as HER2, with a potential dependent expression on eIF4A [7].

Evidence has shown the B-Raf cascade could upregulate the anti-apoptotic proteins Bcl-xL and XIAP [33]; however, in our conditions, the opposite response was observed, suggesting an adaptation that contributed to the development of chemoresistance. Alternate mechanisms for the variant BT474LapRV2 cells are different from the canonical pathway, considering the results shown in Figure 7, showing down-regulated MAPK signaling in the chemoresistance cells. These observations of the down-regulation of Erk and B-Raf targets in BT474^LapRV2^ cells could explain the cellular insensitivity to inhibitors of MEK1/2 previously reported [46].

In BT474^LapRV2^ cells, we observed an NF-κB down-regulation under the joint treatments of Lap and Cry (Figure 9B,C). This phenomenon is consistent with the cell viability results. Our data support a connection between chemoresistance and the down-regulation of MAPK-NF-κB signaling. Indeed, we documented an association among the levels of the NF-κB with the PKCε expression in BT474^LapRV2^ and BT474 cells (Figure 9).

In the human rhabdomyosarcoma model, Cry modulates the mTORC1 pathway by inhibiting p-p70S6K1 and 4EBP1 [47]. Notwithstanding, our data suggest that Cry treatment was insufficient to reverse chemoresistance due to compensatory mechanisms that must be activated, potentially by stabilizing eIF4A and the NF-κB down-regulation.

Other adaptive mechanisms, such as the induction of calcium targets, should contribute to chemoresistance. Indeed, transformed cells remodel intracellular calcium homeostasis to support malignant behavior [26,48] and specialized proteins maintain intracellular calcium homeostasis [24,25], such as PMCA and SERCA. Based on our results, responses mediated by pumps PMCA1/4 and SERCA2 can regulate intracellular calcium concentrations on BT474^LapVar2^ cells despite Lap and Lap plus Cry treatments (Figure 8), indicating the induction of an optimized response in the management of intracellular calcium and potentially inhibiting cellular death. As PMCA2 reports, overexpression lowers intracellular calcium and protects cancer cells from apoptosis [49].

Moreover, to broaden the characterization of the protein modulators of calcium, we assessed the role of PMCA1 and PMCA4 in breast cancer patients’ prognosis databases. Therefore, we evaluated the role of PMCA expression levels in HER2+ breast cancer patients using the Kaplan–Meier Plotter database (https://kmplot.com/analysis/, accessed 12 January 2025). The data showed a marked relationship between high expression of PMCA1/4 and a lower overall survival; as a gene reference, we used *PMCA2* (Figure S6).

Our data indicate a relevant role for target proteins such as PMCA1/4, being a potential therapeutic strategy that involves a selective inhibition of PMCA1/4, aiming to disrupt calcium extrusion and elevate intracellular calcium levels. This proposal is supported by prior studies in which PMCA1/PMCA4 inhibition using specific small molecules was shown to impair calcium extrusion, sensitize breast cancer cells to apoptosis, and reduce tumor cell viability [50,51]; these findings highlight PMCA as a promising therapeutic target, and their inhibition may serve as a viable strategy to overcome calcium-dependent lapatinib resistance in HER2+ breast cancer; however, further research is needed to validate their efficacy.

The role of SERCA2 is more relevant in BT474 cells, considering that PMCA1/4 levels decreased significantly; results suggest SERCA2 is a late-response condition in BT474 parental cells to maintain calcium homeostasis. Indeed, increasing intracellular calcium with an ionophore treatment inhibited HER2 signaling and promoted its internalization and ubiquitination [26]. These suggest that increased calcium levels under treatment with Lap and Cry in BT474 cells is associated with the down-regulation of HER2 signaling and reduced cell viability. Although the data propose an optimized management of calcium levels in the variant BT474^LapRV2^ cells, the impact of cholesterol on the stability of PMCAs and SERCA2 has also been considered [25,35]. To enhance our understanding of Cry treatment, we evaluated the effect of increasing concentrations (0–9 μM); results indicate that Cry treatment is not enough to modify the expression of these pumps (Figure S5). This is due to compensatory cellular responses associated with the function of calcium pumps such as PMCA1/4 and SERCA2, which can regulate intracellular calcium concentrations and maintain cell viability.

Other factors, such as the dysregulation of apoptotic pathways, must contribute to chemoresistance. In this regard, CDK4/6 and PI3K/AKT/mTOR inhibitor therapies have changed patients’ prognoses, greatly benefiting progression-free survival [52]. Notwithstanding, as described in the BT474^LapRV2^ variant, mechanisms such as those adopted in these cells, with intrinsic down-regulation of mTOR and MAPK kinase pathways, may represent an obstacle in therapies.

In the future, we will establish an in vivo model employing the BALB/c mouse model, which is commonly used in cancer research because it is immunocompetent and tends to develop mammary tumors spontaneously in aged mice. This model is essential for understanding tumor development and progression mechanisms and assessing therapeutic interventions. In a parallel project, we are characterizing the impact of dyslipidemia in the chemoresistance acquisition; therefore, we are developing this project, “Low-density lipoproteins (LDL) promote lapatinib chemoresistance through metabolism dysregulation of drug and lipid targets in HER2+ breast cancer” (in process). Likewise, our team is working closely with healthcare institutions in northwestern Mexico to collect clinical data.

On the other hand, Cry is a molecule isolated from natural sources and represents the tip of the iceberg; other opportunities for phytochemicals could be located at the base of the iceberg. The dual treatment strategy could favor the effectiveness of therapeutics [53]. For instance, in MDR1-overexpressing colorectal cancer cells, degraders targeting either the kinases MEK1/2 or the oncogenic mutant GTPase KRASG12C synergized with the Lapatinib have been evaluated [46], and PI3K/AKT/mTOR inhibitors such as Copanlisib have been shown to restore lapatinib sensitivity in BC [15] and gastric cancer cell lines [54] by counteracting pathway overactivation. This evidence positions dual regulation as an optimized strategy in breast cancer chemoresistance.

Beyond cryptotanshinone, other natural compounds have demonstrated the potential to overcome lapatinib resistance. One example is berberine, which can be isolated from several medicinal plants. Berberine inhibits the Nrf2 pathway, promoting increased reactive oxygen species (ROS) and inducing apoptosis in chemoresistant cancer cells [41]. Indeed, studies from our laboratory indicate the presence of a synergistic effect between auraptene and tamoxifen metabolites in a resistant ER+ breast cancer model [53]. Another promising compound is 1′-Acetoxychavicol acetate (ACA) primarily derived from *Zinger officinale.* ACA targets key survival proteins such as BCL2, leading to apoptosis in resistant cancer cells [55].

Our research team is expanding its focus on characterizing natural molecules with therapeutic potential. Currently, we are conducting a review that addresses the role of Cryptotanshinone as an adjutant treatment in both sensitive and chemoresistant cancers (in process).

## 4. Materials and Methods

### 4.1. Reagents and Antibodies

Lapatinib (Lap) (SML2259), Cryptotanshinone (Cry) (C5624), and MTT (Cat. M5655) were purchased from Sigma-Aldrich (St. Louis, MO, USA). Monoclonal antibodies anti-ERα (sc-8002), anti-HER2 (sc-33684), anti-pHER2-^Tyr−124^ (sc-81507), anti-STAT1 (sc-464), anti-PDCD4 (sc-376430), anti-eIF4AI/II (sc-377315), anti-eIF4E (sc-9976), anti-p70S6Kα (sc-8418), anti-p-p70S6Kα (sc-8416), anti-β-actin (sc-47778), and anti-Bcl-xL (sc-8392) were purchased from Santa Cruz Biotechnology (San Diego, CA, USA). Rabbit polyclonal anti-eIF4AI (Cat. 2490) was from Cell Signaling Technology (Beverly, CA, USA). Secondary antibodies (Cat. 31430) were purchased from Thermo Scientific (Somerset, NJ, USA).

### 4.2. Cell Lines and Cell Culture

The BT474 human breast cancer cells (ATCC), BT474^LapRV1^, and BT474^LapRV2^ cell variants were cultured in Dulbecco’s Modified Eagle’s medium (DMEM) supplemented with 10% (*v*/*v*) fetal bovine serum (FBS), penicillin (100 U/mL), streptomycin (100 μg/mL), and amphotericin B (0.25 μg/mL) in a 5% CO_2_ incubator at 37 °C. BT474^LapRV1^ and BT474^LapRV2^ cell variants were cultured continuously with 0.3 and 0.25 μM of Lap, respectively.

### 4.3. Generation of the Lapatinib-Resistant BT474 Cell Line Variants (BT474^LapRV1^ and BT474^LapRV2^)

We developed chemoresistant cellular variants to provide an overall perspective of the chemoresistance mechanisms, considering that widely used clinical HER2 TKIs, such as lapatinib and neratinib, have several drawbacks [56].

To obtain the BT474^LapRV1^ cell line, BT474 human BC cells were cultured with an initial treatment of 1 μM of Lap and maintained at this concentration for 16 days until the monolayer density of the surviving cells was ~80%. Cells were harvested and plated 48 h before the second treatment with Lap 0.2 μM. After 80 days under treatment, the surviving cells’ monolayer density reached ~80%. Finally, cells were harvested and plated 48 h before the third treatment with Lap 0.3 μM. After 11 days under treatment, the surviving cells’ monolayer density was ~80%. A second protocol was developed to generate the BT474^LapRV2^ cell line. BT474 cells were cultured with an initial treatment of Lap 0.1 μM for 37 days, 0.2 μM for 19 days, and 0.25 μM for 11 days. We used these procedures, considering the Lapatinib treatment scheme [57].

BT474^LapRV1^ and BT474^LapRV2^ cell lines were expanded and stored in liquid nitrogen. A parental cell line was grown and denominated BT474. The passages necessary to generate the resistant cell-line variants were identical for the three cellular variants.

### 4.4. Cell Viability Assay

Cells were plated at a density of 2 × 10^4^ cells/well in 96-well plates 48 h before the assay. Cells were incubated with doses of Lap corresponding with specific treatment and Cry for 48 h. Later, the cells were incubated with MTT (0.5 mg/mL) for 2 h. The medium was removed, and the formazan dye crystals were solubilized with 150 μL of acid isopropanol. Absorbance was measured at 590 nm wavelength. The growth percentage was calculated using the number of control cells with vehicles as 100% at 48 h.

### 4.5. Colony Formation Assay

Single-cell suspensions were prepared by trypsinization, followed by cell counting with a hemocytometer. The cells were seeded into 20 mm plates at ~5000 cells/plate and treated with indicated doses of Lap for 22 days. The colonies were stained with MTT reaction for 2 h. The images were captured using a ChemiDoc XRS+ System (Bio-Rad; Hercules, CA, USA). However, under Lap treatments, the resistant variants showed small but Lap-resistant colonies, while in the parental, all cells died. In this context, counting the colonies was unnecessary, but we tried it with an automated system, and the data were inaccurate.

### 4.6. Western Blot Assays

Cells were seeded at 2 × 10^5^ cells/dish density in 20 mm plates. After the corresponding treatment, cells were lysed with lysis buffer (150 mM NaCl, 1% Triton X-100, 0.5% NP40, 1 mM ethylenediaminetetraacetic acid, 10 mM Tris, pH 7.4) and 1× Complete Mini Protease Inhibitor Cocktail (Roche Diagnostics, Branchburg, NJ, USA) for 30 min at 4 °C, and the supernatants were collected by centrifugation. Briefly, an equal amount of protein (13 μg/lane) was resolved on an SDS-8% (*w*/*v*) polyacrylamide gel. Proteins were transferred to a PVDF membrane (Millipore, Burlington, MA, USA). Membranes were blocked (1 h at 25 °C) with TBS1X-Tween 0.05% containing nonfat milk (5%; *w*/*v*), then incubated overnight at 4 °C with the corresponding primary antibodies, followed by 2 h incubation with secondary antibodies conjugated to horseradish peroxidase (HRP). Protein was detected by Immobilon Western kit (Immobilon Western from Millipore, Burlington, MA, USA). Signal intensity was determined densitometrically using Image Lab software, version 5.1, from Bio-Rad Laboratories (Hercules, CA, USA). Anti-β-actin and anti-GAPDH were used as loading control.

### 4.7. Small Interfering RNA (siRNA)

Cellular cultures were seeded at a density of 2 × 10^5^ in 20 mm plates and incubated overnight in a basal growth medium without antibiotics. eIF4AI siRNA (h) (sc-40554) and control siRNA-A (sc-37007) were purchased from Santa Cruz Biotechnology (Santa Cruz, CA, USA). Transfection was performed according to the manufacturer’s protocol. The results were confirmed by the Western blot of eIF4A.

### 4.8. eIF4AI Overexpression and Purification

The gene encoding for *eIF4AI* cDNA (NM 001416.4) under the control of CMV-promotor in a pcDNA3.1(+) vector was acquired under the project ID: U533ZGE280-1, clone ID: HP4425A (GenScript, Piscataway, NJ, USA). The plasmid was transformed into *Escherichia coli* cell strain Rosseta-Star (Novagen, Madison, WI, USA), harboring the pET19b-*eIF4AI* plasmid. Cultures were grown at 37 °C in 2× YT medium complemented with ampicillin (100 µg/mL) to reach an optical density (OD_600_) of 0.6, followed by IPTG (1 mM) induction and further incubation for 12–20 h at 37 °C. After harvesting by centrifugation, cells were disrupted by sonication; the supernatant was obtained and washed with 50 mM NaH_2_PO_4_, 300 mM NaCl, and 10 mM Imidazole. Purification was performed by affinity chromatography (Ni-NTA Agarose resin QIAGEN, Hilden, Germany). The His-tag was removed with PPS, and the purified eIF4A was dialyzed against PBS and treated with PPS following the manufacturer’s protocol. PPS was eliminated by glutathione Sepharose (GE, Chicago, IL, USA). eIF4A was characterized by SDS-PAGE gel with Coomassie blue staining and WB.

### 4.9. Interaction Assay

Protein–ligand interaction assays were performed. Fluorescence measurements were performed with a Cary Eclipse fluorometer, using the following parameters: a scan from 250 to 350 nm at 25 °C in a synchronous mode. We evaluated the protein–ligand interaction under eIF4A (12 µM) and Cry (0–50 μM). Solutions were homogenized and incubated for 5 min at 25 °C; measurements were performed in a quartz cell with a path length of 1.0 cm and a 500 µL volume.

### 4.10. Molecular Docking

The structures of ligand molecules were obtained from the PubChem database [58], Cry (CID 160254), and fulvestrant (CID 104741). The PDB three-dimensional structure of ERα at 1.9 Å resolution (3ERT) and HER2+ at 2.25 Å resolution (3PP0) were characterized. The protein structures were prepared by removing water and small molecules, leaving only the protein structure. The ligand and receptor were 3D-protonated, and energy minimization was performed using Molecular Operating Environment (MOE) software version 2022.02 with default parameters under the AMBER99 force field. The ligand generates different conformations using a stochastic search in MOE default parameters.

Molecular docking was set as the default parameter for MOE software, and the pre-conformations were employed. To analyze docking results, MOE identifies salt bridges, hydrogen bonds, hydrophobic interactions, sulfur-LP, cation-π, and solvent exposure, and gives the S score (MOE). Ligand interactions with target proteins were predicted based on the S score.

### 4.11. Intracellular Calcium Quantification

Cells were processed as previously described [35], and calcium levels were calculated according to Patel et al. (2003) [59]. After treatments, cell variants were washed with PBS IX and incubated at 37 °C with 1.5 µM Fura-2/AM in the opti-MEM medium for 75 min. Subsequently, cells were washed with PBS and incubated for 20 min at 25 °C. Cells were washed with PBS, trypsinized, inactivated, and finally washed with PBS. Fluorescence measurements were carried out at 340 and 380 nm excitation wavelengths and a 510 nm emission wavelength, employing a Cary Eclipse fluorescence spectrophotometer (Santa Clara, CA, USA). R_max_ was obtained by adding Triton X-100 (0.035%) and R_min_ with the EGTA (4.5 mM) incubation.

### 4.12. Zymogram Assays

BT474 variant cultures were incubated under Cry and Lap treatments, and the conditioned medium was collected. Volumes of 40 µL non-heated conditioned medium samples were mixed with 5× sample buffer (0.313 M Tris pH 6.8, 10% SDS, 50% glycerol, and 0.05% bromophenol blue) and loaded on 8% polyacrylamide gels copolymerized with gelatin (1% *w*/*v*). Gels were rinsed twice with 2.5% Triton X-100 and then incubated in a development buffer (50 mM Tris–HCl pH 7.4, 10 mM CaCl_2_, and 0.02% NaN_3_) for 40 h at 37 °C. Gels were fixed and stained with 0.25% Coomassie brilliant blue G-250. Proteolytic activity was detected as clear bands against the background stain of the unprocessed substrate. MDA-MB-231 cells were used as a positive control.

### 4.13. Kaplan–Meier Plotter Analysis

We use the KM-plot database (Győrffy, B) to evaluate the implication of PMCA1 (gene: *ATP2B1*), PMCA2 (gene: *ATP2B2*), and PMCA4 (gene: *ATP2B2*) expression and its relationship with survival HER2+ patients. We selected the TCGA-RPPA database for the KM analysis. Specifically, we used the mRNA-seq tool (https://kmplot.com/analysis/; accessed on 20 January 2025).

### 4.14. Statistical Analysis

Results are expressed as the mean ± SD of at least three independent experiments. The IC_50_ values for treatments were calculated using nonlinear regression (curve fit) by log [Lap or Cry] vs. a normalized response–variable slope. Statistical analysis was carried out using one-way ANOVA followed by Dunnett’s Multiple Comparison test or Turkey’s Multiple Comparison test. All statistical analysis was performed using PRISM Software (Version 8.0; GraphPad, San Diego, CA, USA).

## 5. Conclusions

We developed TKI-lapatinib chemoresistant cellular variants (BT474^LapRV1^ and BT474^LapRV2^) and characterized the pathways that define their chemoresistance. We identified that the PDCD4-p70S6Kβ axis is associated with Lapatinib chemoresistance, and we described the impact of Cry on the potential chemosensitivity. Down-regulation of cell survival pathways RAF-MEK-ERK and concomitant NF-κB were identified as a potential fingerprint condition for chemoresistance. Results indicated the development of the adaptive mechanism through optimal calcium management, determined by increased expression of calcium pumps PMCA1/4 and SERCA2.

## Figures and Tables

**Table 1:** Effect of Cryptotanshinone (Cry) treatment on the Lapatinib activity in cellular variants.

*Cellular Variant*	*BT474*	*BT474^LapRV1^*	*BT474^LapRV2^*
IC_50_ Lap	3.16 ± 0.06	34.72 ± 0.11	23.3 ± 0.13
IC_50_ Lap + Cry	2.2 ± 0.04	48.28 ± 0.09	36.04 ± 0.07

The Cry dose was 9.34 µM, corresponding to IC_50_ of BT474 cells.

## Data Availability

Data are contained within the article.

## Supplementary Materials

The following supporting information can be downloaded at https://www.mdpi.com/article/10.3390/ijms26083763/s1.
