# Supplementary material for: Lapatinib-Resistant HER2+ Breast Cancer Cells Are Associated with Dysregulation of MAPK and p70S6K/PDCD4 Pathways and Calcium Management, Influence of Cryptotanshinone
Source: Int J Mol Sci. 2025 Apr 16;26(8):3763. doi: 10.3390/ijms26083763 (PMC12027730; doi:10.3390/ijms26083763)

Lapatinib-Resistant HER2+ Breast Cancer Cells Are Associated with Dysregulation of MAPK and p70S6K/PDCD4 Pathways and Calcium Management, Influence of Cryptotanshinone

Jorge Hernández-Valencia [1,2,†], Ruth García-Villarreal [1,2,†], Manuel Rodríguez-Jiménez [1,2], Alex Daniel Hernández-Avalos [1,2], Ignacio A. Rivero [3], José Luis Vique-Sánchez [4], Brenda Chimal-Vega [1,2,5], Angel Pulido-Capiz [1,2] and Victor García-González [1,2,5,*]

1 Departamento de Bioquímica, Facultad de Medicina Mexicali, Universidad Autónoma de Baja California, Mexicali 21100, Baja California, Mexico; jhernandezv@cinvestav.mx (J.H.-V.); ruth.garcia@uabc.edu.mx (R.G.-V.); srodriguez5@uabc.edu.mx (M.R.-J.); alex.hernandez11@uabc.edu.mx (A.D.H.-A.); brenda.chimal@uabc.edu.mx (B.C.-V.); pulido.angel@uabc.edu.mx (A.P.-C.)

2 Laboratorio Multidisciplinario de Estudios Metabólicos y Cáncer, Facultad de Medicina Mexicali, Universidad Autónoma de Baja California, Mexicali 21100, Baja California, Mexico

3 Centro de Graduados e Investigación en Química, Tecnológico Nacional de México, Instituto Tecnológico de Tijuana, Tijuana 22510, Baja California, Mexico; irivero@tectijuana.mx

4 Centro de Ciencias de la Salud Mexicali, Universidad Autónoma de Baja California, Mexicali 21000, Baja California, Mexico; jvique@uabc.edu.mx

5 Centro de Innovación e Investigación en Salud (CIIS), Universidad Autónoma de Baja California, Mexicali 21000, Baja California, Mexico

* Correspondence: vgarcia62@uabc.edu.mx

† These authors contributed equally to this work.

Supplementary Figure S1. Down-regulation of eIF4AI in BT474 and BT474^{LapRV2} cells through siRNA. Cells were transfected with siRNA sequences against eIF4AI (siRNA-eIF4AI) and non-transfected cells as control (Ctrl). **(A)** Images of parental and BT474^{LapRV2} cells 24 h post-transfection; scale bar, 10X. **(B)** eIF4AI and PDCD4 were assessed by western blot using specific antibodies. **(C)** Densitometric analysis of eIF4AI after β-actin normalization.

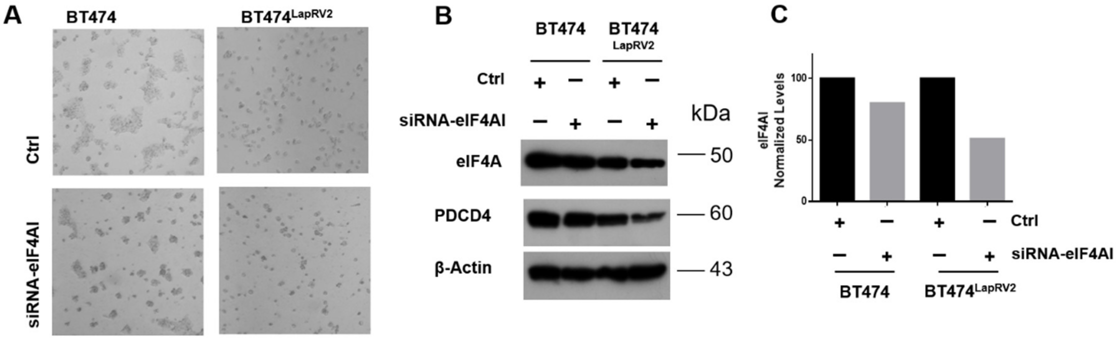

Supplementary Figure S2. Expression of ERα is down-regulated in BT474^{LapRV1} and BT474^{LapRV2} chemoresistant cells. **(A)** Immunoblot of ERα. During the generation of the resistant cells, whole cell lysates were prepared and subjected to immunoblot analyses with the indicated antibody. **(B)** Densitometric analysis of ERα after β-actin normalization. Results are presented as the mean of three independent experiments ± SD. ** $p < 0.01$; *** $p < 0.005$.

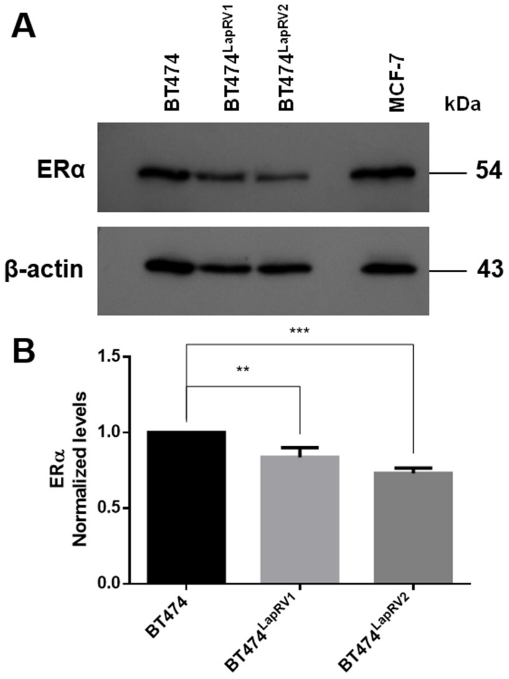

Supplementary Figure S3. MMP-9 activity in BT474 and BT474^{LapRV2} cells on the supernatant medium under the Lap (0.3 µM) and Cry (9 µM) joint treatment. MDA-MB-231 (TNBC) cell supernatant media was used as a positive control.

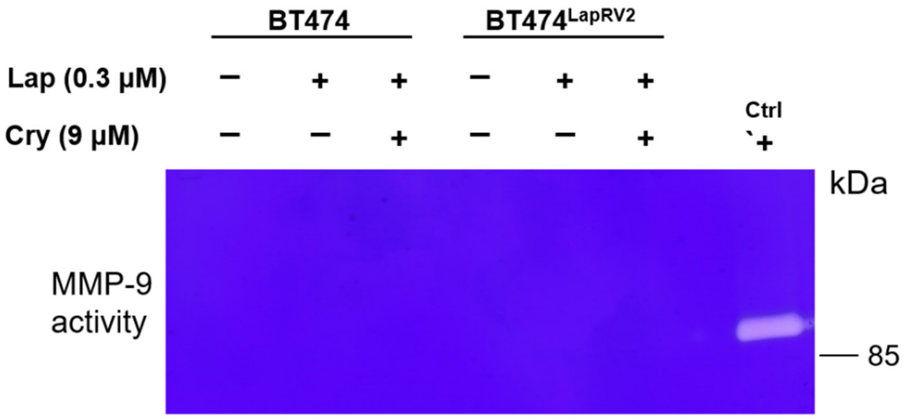

Supplementary Figure S4. Molecular interactions of ERα with Cry and Fulvestrant molecules. **A)** The ligand interaction of Cry shows the ERα residues and the type of interaction. **B)** The ligand interaction of Fulvestrant shows the ER residues and the type of interaction. The PDB three-dimensional structure was used at 1.9Å resolution (3ERT).

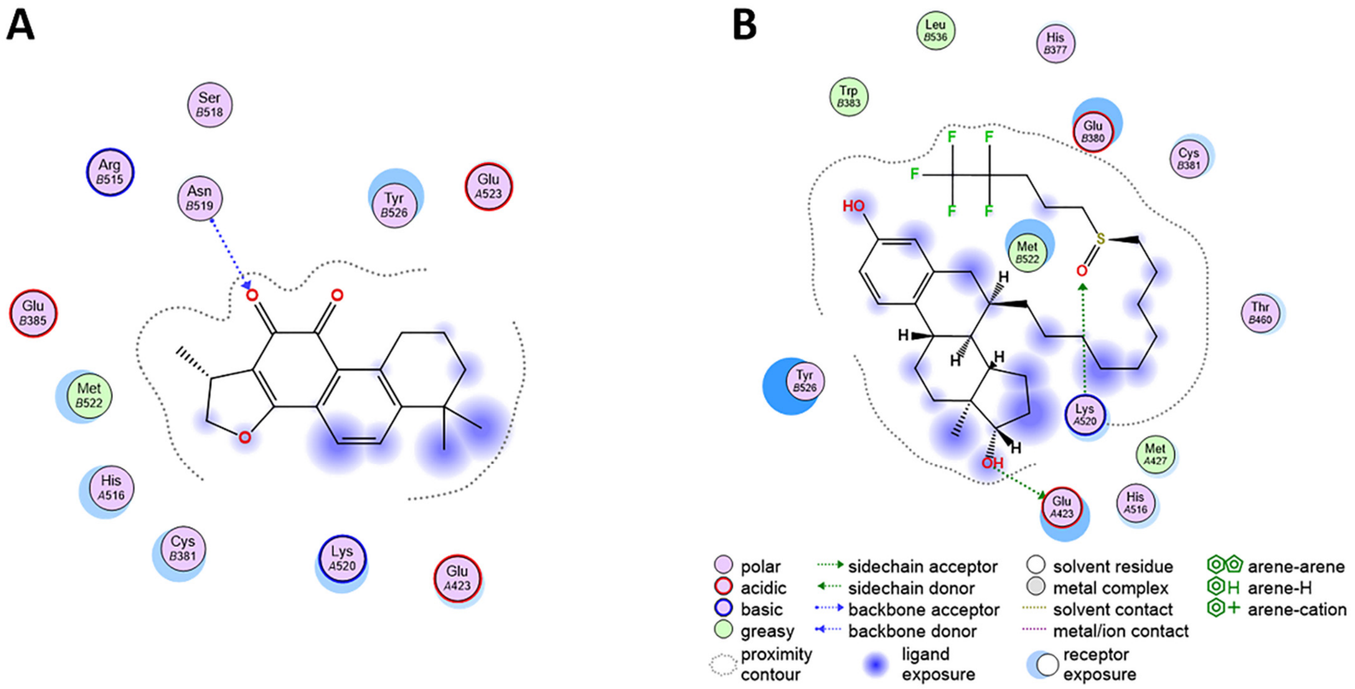

Supplementary Figure S5. Effect of Cryptotanshinone treatment (0-9 µM) on the PMCA1/4 and SERCA2 expression in BT474 cells. GAPDH was used as a loading control.

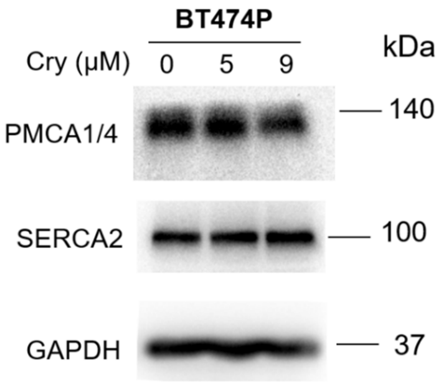

Supplementary Figure S6. Overall survival plot for low and high expression of PMCA1 (gene: ATP2B1) (A), PMCA4 (gene: ATP2B4) (**B**) and SERCA2 (gene: ATP2A2) (**C**) in HER2+ breast cancer patients. For this analysis, we selected the TCGA-RPPA database for the KM analysis. Specifically, we used the mRNA-seq tool.

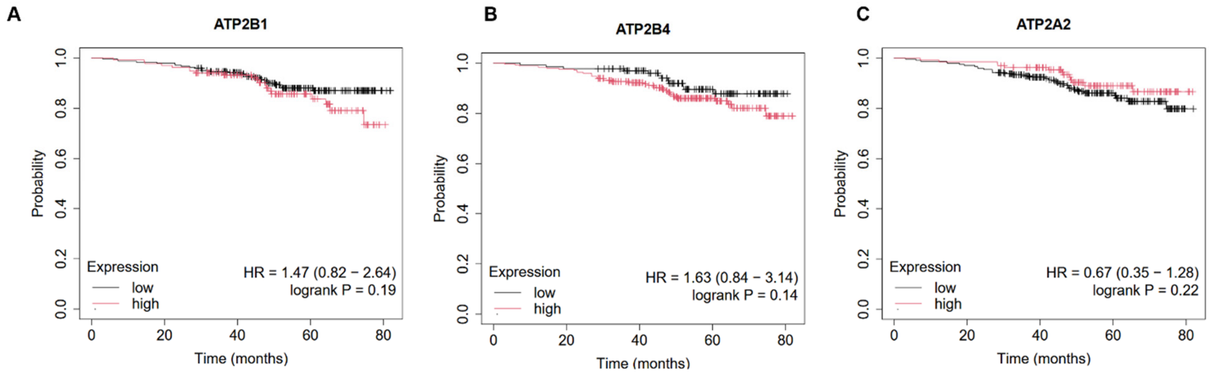

Supplement: Supplementary file 1 [file ijms-26-03763-s001.zip › ijms-3520645-supplementary.pdf]
